# 1,635 Endoscopic submucosal dissection cases in the esophagus, stomach, and colorectum: complication rates and long-term outcomes

**DOI:** 10.1007/s00464-012-2555-2

**Published:** 2012-10-06

**Authors:** Takashi Toyonaga, Mariko Man-i, James E. East, Eisei Nishino, Wataru Ono, Tomoomi Hirooka, Chie Ueda, Yoshinori Iwata, Takeshi Sugiyama, Toshio Dozaiku, Takashi Hirooka, Tsuyoshi Fujita, Hideto Inokuchi, Takeshi Azuma

**Affiliations:** 1 Department of Endoscopy, Kobe University Hospital, 7-5-1 Kusunoki-cho, Chuo-ku, Kobe, Hyogo 650-0017 Japan; 2 Frontier Medical Science in Gastroenterology, Kobe University School of Medicine, Kobe, Hyogo Japan; 3 Translational Gastroenterology Unit, John Radcliffe Hospital, Oxford, United Kingdom; 4 Pathology, Kishiwada Tokushukai Hospital, Kishiwada Osaka, Japan; 5 Gastroenterology, Kishiwada Tokushukai Hospital, Kishiwada Osaka, Japan; 6 Gastroenterology, Fuchu Hospital, Izumi Osaka, Japan; 7 Gastroenterology and Hepatology, Hyogo College of Medicine, Nishinomiya Hyogo, Japan; 8 Gastroenterology, Sakibana Hospital, Izumi Osaka, Japan; 9 Gastroenterology, Hyogo Cancer Center, Akashi Hyogo, Japan

**Keywords:** Endoscopic submucosal dissection, Complication, Postoperative, Prognosis, Neoplasms

## Abstract

**Background:**

Endoscopic submucosal dissection (ESD) enables en bloc resection of early gastrointestinal neoplasms; however, most ESD articles report small series, with short-term outcomes performed by multiple operators on single organ. We assessed short- and long-term treatment outcomes following ESD for early neoplasms throughout the gastrointestinal tract.

**Methods:**

We performed a longitudinal cohort study in single tertiary care referral center. A total of 1,635 early gastrointestinal neoplasms (stomach 1,136; esophagus 138; colorectum 361) were treated by ESD by single operator. Outcomes were complication rates, en bloc R0 resection rates, and long-term overall and disease-specific survival rates at 3 and 5 years for both guideline and expanded criteria for ESD.

**Results:**

En bloc R0 resection rates were: stomach: 97.1 %; esophagus: 95.7 %; colorectum: 98.3 %. Postoperative bleeding and perforation rates respectively were: stomach: 3.6 and 1.8 %; esophagus: 0 and 0 %; colorectum: 1.7 and 1.9 %. Intra criteria resection rates were: stomach: 84.9 %; esophagus: 81.2 %; colorectum: 88.6 %. Three-year survival rates for lesions meeting Japanese ESD guideline/expanded criteria were for all organ-combined: 93.4/92.7 %. Five-year rates were: stomach: 88.1/84.6 %; esophagus: 81.6/57.3 %; colorectum: 94.3/100 %. Median follow-up period was 53.4 (range, 0.07–98.6) months. Follow-up rate was 94 % (1,020/1,085). There was no recurrence or disease-related death.

**Conclusions:**

In this large series by single operator, ESD was associated with high curative resection rates and low complication rates across the gastrointestinal tract. Disease-specific and overall long-term prognosis for patients with lesions within intra criteria after curative resection appeared to be excellent.

Early gastrointestinal tract neoplasia is increasingly recognized and detected by endoscopists, due to improvements in endoscopic image quality and increased education and awareness of these often flat lesions [1]. It is now accepted that even large lesions can be endoscopically curatively managed as long as they are not deeply invasive. Endoscopic mucosal resection (EMR) with or without a cap has been the primary technique for managing these lesion; however, EMR of lesions larger than 20 mm in diameter often results in piecemeal resection, which may lead to inconclusive or incorrect histopathological evaluation and local recurrence.

Endoscopic submucosal dissection has enabled en bloc resection of lesions that are difficult to remove en bloc by conventional endoscopic mucosal resection (EMR) [2–9] regardless of tumor location and size. ESD was introduced for treatment of larger mucosal cancers and slightly invasive submucosal cancers in the upper and lower gastrointestinal tract, which have a very low incidence of lymph node metastasis, making them potentially candidates for endoscopic treatment [10, 11]. ESD is the procedure of choice for early gastric cancer in Japan and is increasingly used for esophageal and colorectal lesions. Experience is growing in the rest of Asia, Europe, and Latin America, but use in the United States is very rare.

Many articles have reported the treatment results of ESD; however, most of them were relatively small series and often were performed by multiple operators. This makes it difficult to assess precisely aspects such as complication and en bloc resection rates, because adverse events are relatively rare and may vary substantially between operators and organs. Importantly, there are few reports about the long-term outcomes of ESD in terms of recurrence rates and survival with an acceptable large dataset and follow-up period.

In this retrospective longitudinal cohort study, we assessed short-term and long-term treatment outcomes for a large number ESDs for early gastrointestinal neoplasms in the esophagus, stomach, and colorectum by single operator. We particularly focus on long-term survival both overall and by organ.

## Patients and methods

A total of 1,659 lesions (stomach 1,145; esophagus 139; colorectum 375) were attempted for ESD, and 1,635 lesions/1,261 patients (stomach 1,136/821; esophagus 138/111; colorectum 361/329) were completed between May 2002 and July 2007 and were analyzed retrospectively. Cases with multiple lesions were included, and patients with recurrence after EMR were included in this study. The study protocol was approved by the Ethics Committee of Kishiwada Tokushukai Hospital. This study has been registered in the University Hospital Medical Information Network Clinical Trials Registry (UMIN-CTR) as number UMIN000006140. The process of treatment, including complications and the possibility of additional surgery because of perforation or the pathologic diagnosis of resected specimens, was clearly explained to all patients, and their written informed consent was obtained. All noncompleted cases underwent surgical treatment.

### Criteria

The criteria for ESD were determined by the endoscopic characteristics and histological findings of biopsy specimens; endoscopic ultrasonography also was performed when the lesion was strongly suspected of submucosal invasion. The criteria in this report were shown in Box 1, which broadly follow Japanese guidelines.

**Box 1:** Criteria in this study

Stomach
Guideline criteria
1. adenoma (ade),
2. mucosal (m)-cancer (ca), differentiated (diff.) type, ly (lymphatic invasion) (−), v (vascular invasion) (−), and ulceration (Ul) (−) and ≤2 cm in size
Expanded criteria
1. m-ca, diff. type, ly(−), v(−), Ul(−) and >2 cm in size
2. m-ca, diff. type, ly(−), v(−), Ul(+) and ≤3 cm in size
3. submucosal (sm) 1-ca (invasion depth <500 μm), diff. type, ly(−), v(−), and <3 cm in size [12, 13]
Extra criteria
Others than guideline criteria or expanded criteria
Esophagus (squamous lesions only)
Guideline criteria
1. low grade intraepithelial dysplasia (LGIN), high grade intraepithelial dysplasia (HGIN)
2. cT1a-epithelium(EP)-ca, 3), cT1a-lamina propria mucosae(LPM)-ca
Expanded criteria
1. cT1a-muscularis mucosa(MM)-ca, ly(−), v(−), inf α, diff. type, ly(−), v(−)
2. cT1b/sm-ca (invasion depth < 200 μm), ly(−), v(−), infiltrative growth pattern, expansive type (inf α), diff. type, ly(−), v(−) [14, 15]
Extra criteria
Others than guideline criteria or expanded criteria
Colorectum
Guideline criteria
1. ade
2. m-ca
Expanded criteria
1. sm-ca (<1000 μm), diff. type, ly(−), v(−)
Extra criteria
Others than guideline criteria or expanded criteria [16]

### Method of ESD procedure

All procedures were performed by one experienced endoscopist (TT) who had conducted more than 2,000 ESDs during a period of 5 years. The outline of the procedure is described hereunder.

### Technical devices

#### Primary endo-knives

We used the FlexKnife (KD-630L, Olympus Optical Co, Ltd, Tokyo, Japan) [5, 8] in the colorectum and short needle knife in the esophagus between May 2002 and June 2005, FlushKnife (DK-2618JN, Fujifilm Optical Co, Ltd, Tokyo, Japan) [17, 18] following June 2005, and IT knife (KD-610L, Olympus) [2, 3] mainly in the stomach and not in the colorectum. FlushKnife is a short needle knife with water jet emitting function, which can dissect severe fibrosis and also add the local injection by knife itself.

#### Ancillary devices

The conventional Needle knife (KD-10Q-1, Olympus), precut Needle knife, the Hook knife (KD-620LR, Olympus) [4], and ST hood (DH-16CR, Fujinon) were used as ancillary devices.

#### Endoscopes

A single channel endoscope (GIF Q 240I for esophagus and stomach, Olympus; CF 240I for colorectum, Olympus) was used with a 4 mm-long, transparent hood to keep a clear operating field.

#### Electrosurgical generator

ICC200, VIO 300D (ERBE Elektromedizin GmbH, Tubingen, Germany) was used as the electrosurgical generator.

#### Injection solution

Saline for the stomach and diluted sodium hyaluronate solution (MucoUp; Johnson & Johnson K.K., Tokyo, Japan) for the esophagus and colorectum was used as local injection into the submucosal layer providing a distinct and long-lasting mucosal elevation [19].

### ESD technique

The lesions were first identified and demarcated using white-light endoscopy, magnifying endoscopy, and chromoendoscopy. Then, marking around the lesions was performed except in the colorectum. Local injection was made using injection needle, and then mucosal incision was performed around the lesion using primary endo-knives. Additional local injection was made using injection needle or Flush knife. Submucosal dissection was performed using primary endo-knives; however, ancillary devices were used combined in difficult cases. Hemostasis and vessel coagulation were practiced using primary endo-knives or hemostatic forceps. The precise procedure has been described elsewhere [17, 18, 20–23].

### Postoperative bleeding

Postoperative bleeding was defined as the decrease of hemoglobin by 2 g/dl caused by hematemesis or melena and the need for hemostatic treatment by endoscopic management.

### Perforation

Immediate perforation was recognized endoscopically and was treated by endoscopic clipping, fasting, and antibiotics. Clinical examination and an erect chest and abdominal x-ray were performed routinely 1 day postprocedure to look for delayed perforation. Delayed perforation was treated by surgical intervention for fear of severe peritonitis caused by enteric bacteria, when the inflammation manifested as localized abdominal pain, leucocytosis, and occasionally fever were uncontrollable by conservative treatment.

### Histopathological assessment

The resected specimens were stretched and fixed onto a rubber plate and immersed in formalin and sectioned serially at 2 mm intervals and subjected to histopathological examination.

### En bloc resection, en blocR0 resection, intra-criteria resection

Resection of the lesion in a single piece was defined as en bloc resection. Resection of lesions in one piece with margins free of the tumor was defined as en bloc R0 resection. The minimum distance required for margin free was 0.5 mm in lateral side and 50 μm in vertical side.

Specimens meeting the above guideline criteria or expanded criteria were considered to represent curative resection with little risk of lymph node metastasis, and described as intra-criteria resection (Box 1), with guideline and expanded criteria.

### Follow-up

Those cases in which after resection, pathology indicated that they continued to be within guideline or expanded criteria for ESD following pathological examination (intra-criteria cases) were examined by periodic surveillance endoscopy to check for recurrence (stomach and esophagus: generally 6 months after ESD, and every 1 year thereafter; colorectum: generally 1 year after ESD, and every 3 years thereafter). We routinely performed systematic chromoendoscopy of the scar site. Biopsy was performed in any case where recurrence was suspected. The cases also are examined by annual thoracic and abdominal computed tomography. The data were collected from the medical records. Incomplete data were investigated from the telephone contact with patients, family, or a request for information from the referring physician.

Patients whose lesions were found to be outside the guideline or expanded criteria for ESD following pathological examination (extra criteria cases) were advised to undergo further surgery. The cases surveyed until June 2009 were defined as the cohort who underwent follow-up, and the flow diagram of patient’s follow-up is shown in Table 1.

**Table 1:** Patient and lesion demographic data, complication rates and resection rates

	Stomach	Esophagus	Colorectum	Combined
No. of lesions	1136	138	361	1635
Age (years)	71	69	68	70
[range]	[31–93]	[43–90]	[20–92]	[20–93]
Sex (M:F)	854:282	113:25	204:157	1171:464
Median tumor size (cm)	13	23	30	17
[range]	[1–105]	[1–72]	[6–158]	[1–158]
Median resected specimen size (cm)	42	45	40	42
[range]	[14–153]	[22–90]	[16–165]	[14–165]
Criteria (%)				
Guideline	733 (64.5)	89 (64.5)	295 (81.7)	1117 (68.3)
Expanded	235 (20.7)	25 (18.1)	29 (8.0)	289 (17.7)
Extra	168 (14.8)	24 (17.4)	37 (10.3)	229 (14.0)
UL (+)	8.4 %	–	–	
	(95/1136)			
[95 % CI]	[6.8–10.0]			
Postoperative bleeding	3.6 %	0.0 %	1.7 %	2.9 %
	(41/1136)	(0/138)	(6/361)	(47/1635)
[95 % CI]	[2.5–4.7]	[0.0–0.0]	[0.4–3.0]	[2.1–3.7]
Perforation	1.8 %	0.0 %	1.9 %	1.7 %
	(21/1136)	(0/138)	(7/361)	(28/1635)
En bloc resection	99.3 %	98.6 %	98.6 %	99.1 %
	(1128/1136)	(136/138)	(356/261)	(1620/1635)
[95 % CI]	[98.8–99.8]	[96.6–100.6]	[97.4–99.8]	[98.6–99.6]
En bloc R0 resection	97.1 %	95.7 %	98.3 %	97.2 %
	(1103/1136)	(132/138)	(355/361)	(1590/1635)
[95 % CI]	[96.1–98.1]	[92.3–99.1]	[97.0–99.6]	[96.4–98.0]
Intra criteria resection	84.9 %	81.2 %	88.6 %	85.4 %
	(964/1136)	(112/138)	(320/361)	(1396/1635)
[95 % CI]	[82.8–87.0]	[74.7–87.7]	[85.3–91.9]	[83.7–87.1]

### Evaluated parameters

The retrospectively evaluated data included the en block resection rate, en bloc R0 resection rate, rates of cases under the guideline criteria and expanded criteria, intra-criteria resection rate, tumor diameter, resected specimen diameter, macroscopic type, location, postoperative bleeding rate, and perforation rate. All procedures were recorded on videotape and parameter, such as perforation was noted for evaluation. Data are reported according to the STROBE guidelines for reporting observational studies [24].

### Statistics

Values were presented as medians. Independent continuous variables were compared by the Mann–Whitney test, and categorical variables were compared by the χ^2^ test or Fisher’s exact test using Statview version 5.0.

Data for the long-term outcomes were calculated using the Kaplan–Meier method and analyzed by the log-rank test. All *p* values were two-sided, and *p* values ≤0.05 was considered to be statistically significant.

## Results

### En bloc resection rates, intra criteria resection rates and complication rates

Reported lesions treated are 1,136 gastric, 138 esophageal, and 361 colorectal. ESD experience was first gained in the stomach, next in colorectum, and then in the esophagus as the operator became more experienced. En bloc resection rates, intra criteria resection rates and complication rates are presented in Table 1.

### Long-term follow-up

The follow-up for patients until June 2009 are presented in Table 2. The follow-up for lesions within guideline criteria and expanded criteria was a median of 53.4 (range, 0.07–98.6) months. Follow-up periods less than 23.6 months were caused by death from other cancers and unrelated diseases.

**Table 2:** Patients follow-up rate to June 2009

	Guideline criteria	Expanded criteria	Multiple lesions*	Guideline criteria + Expanded criteria + Multiple lesions*	Total patients	Follow-up rate
Stomach	387	132	153	672	701	95.9 % (672/701)
Esophagus	56	16	16	88	89	98.9 % (88/89)
Colorectum	226	25	18	269	295	91.2 % (269/295)
Organ-combined	669	173	187	1029	1085	94.8 % (1,029/1085)

* Multiple lesions consisted of those in guideline criteria or expanded criteria

**Fig. 1:**
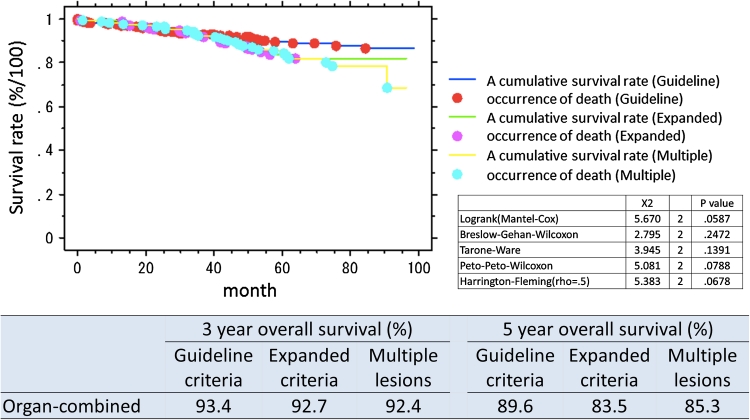
Survival curves following ESD, split into guideline criteria, expanded criteria, and multiple lesions, organs combined

**Fig. 2:**
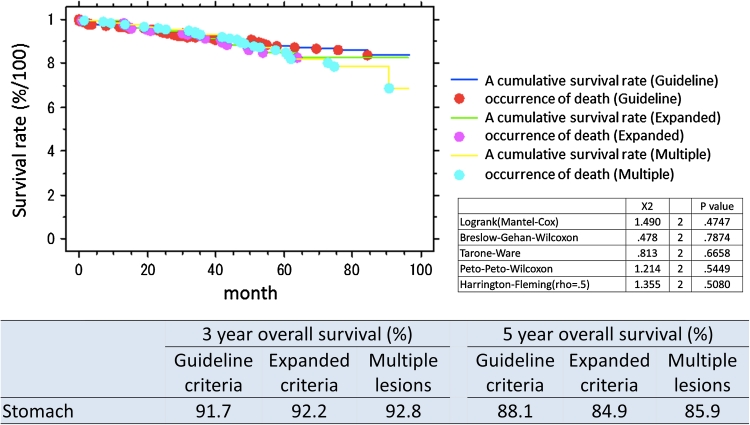
Survival curves following ESD, split into guideline criteria, expanded criteria, and multiple lesions, stomach

**Fig. 3:**
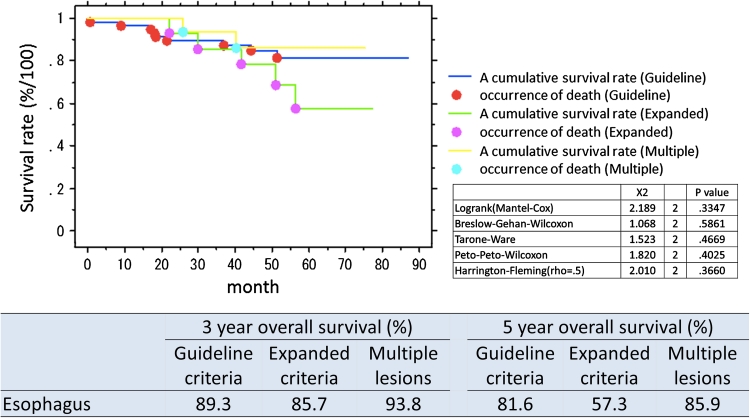
Survival curves following ESD, split into guideline criteria, expanded criteria, and multiple lesions, esophagus

**Fig. 4:**
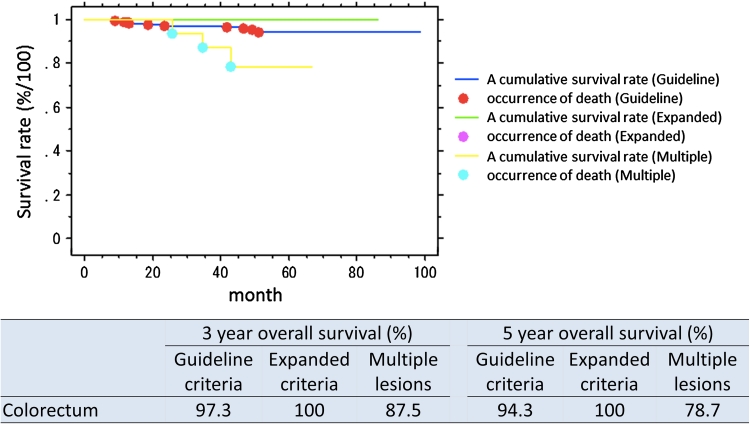
Survival curves following ESD, split into guideline criteria, expanded criteria, and multiple lesions, colorectum

The organ-combined, 3 year overall survival rate for patients with lesions within guideline criteria was 93.4 % and for expanded criteria 92.7 %. Five-year overall survival rate was 88.1 % for guideline criteria lesions and 84.6 % for expanded criteria lesions. The organ-specific survival is presented in Table 3. Survival curves overall and by organ and lesions type (guideline criteria/expanded criteria/multiple lesions) are presented in Figs. 1, 2, 3, and 4. There was no significant difference in prognosis between guideline criteria and expanded criteria in stomach, esophagus, and colorectum, and there was no recurrence or disease-related death.

**Table 3:** 3-Year and 5-year overall survival rates in organ-combined, stomach, esophagus, and colorectum

	3-Year overall survival (%)			5-Year overall survival (%)		
	Guideline criteria	Expanded criteria	Multiple lesions	Guideline criteria	Expanded criteria	Multiple lesions
Organ-combined	93.4	92.7	92.4	89.6	83.5	85.3
Stomach	91.7	92.2	92.8	88.1	84.6	85.9
Esophagus	89.3	85.7	93.8	81.6	57.3	85.9
Colorectum	97.3	100	87.5	94.3	100	78.7

## Discussion

Endoscopic submucosal dissection (ESD) has been widely accepted in Japan, because it has enabled the en bloc resection of early gastrointestinal neoplasms, and it is expected to be a curative but less radical treatment than conventional surgical treatment. This study represents the largest ESD cohort yet reported, leading to narrow confidence intervals for estimates, and the possibility to assess for differences in complications and en bloc resection rates between organs of the gastrointestinal tract to guide clinicians in assessing risks and benefits of ESD when appropriate for their patients. It also provides robust long-term follow-up data to guide likely prognosis following ESD for both organ- and lesion-specific indications.

### Main findings

The en bloc R0 resection rate and intra criteria resection rate were similar and good in stomach, esophagus, and colorectum. Complication rates were low raging from 0 to 3.6 %. There were no complications (postoperative bleeding or perforation) in the esophagus; however, this may have been affected by prior experience in stomach and colorectum. Disease-specific survival rates following en bloc R0 resection were 100 %, probably reflecting the lack of recurrence noted. Long-term survival overall also was good, with no differences between organs.

Follow-up rate was high, in excess of 90 %, and follow-up period was a median of 4.4 years. There was no significant difference in prognosis between guideline criteria, expanded criteria, and multiple lesions which consist of guideline or expanded criteria lesions. There was also no significant difference of prognosis in these three groups by organ or lesion criteria with no disease-specific death or recurrence, suggesting that the assumption that ESD is an appropriate and curative but less radical approach than surgery is true.

### Comparisons with other studies

The overall and by organ en bloc resection rates are equal to or better than other series of ESD, where rates varied between 70 and 90 % (stomach 76–96 %; esophagus 95–100 %; colorectum 77–98.6 %), and the perforation rates and perforation rates by organ also are similar to or lower than other series (bleeding rate/perforation rate: stomach 3–7 %/1–12 %; esophagus 0–1 %/6–7 %; colorectum 1–12 %/1–6 %) [15, 25–27].

A prospective, single-arm, multicenter, phase II trial for the prognosis of expanded criteria in stomach has started to address this, and the result will be available in 5 years [28]. Other prospective, multicenter studies looking at expanded criteria in the esophagus and colorectum are being planned. Pending those study outcomes, these results suggest that patients with lesions within guideline and expanded criteria treated by high-quality ESD are likely to have excellent long-term prognosis.

### Clinical implications

These data show that with careful technique and experience, ESD can be a safe procedure with low complication rates, high disease-specific cure rates, and good long-term survival. This should encourage clinicians to select ESD performed by experienced operators as a potential or even preferred treatment option for early gastrointestinal tract neoplasia. Paradoxically, although the esophagus is considered technically challenging due to thin wall and narrow lumen, in fact complications were lowest here.

### Limitations

Most importantly, this was a single-operator, retrospective study, meaning that the results may not be generalizable; however, the majority of the data, such as lesion size and bleeding, was collected in a systematic way at the time the cases were performed, making the dataset relatively robust. Caution should be used in applying the data to non-Asian populations. The long-term survival rates may not be transferable to operators with lower en bloc R0 resection rates.

### Future directions

ESD is evolving as a technique with little data from mature cohorts to assess its success. The current data support the use of ESD; however, the procedure remains technically demanding and time-consuming and complications continue to occur even after 1,000 procedures. More precise criteria on which lesions justify the risk and effort of ESD compared with EMR will be needed. Balanced against this is that development of endo-knives may make the procedure faster and safer, particularly the ability to inject fluid and cut within the same device. Improved hemostatic capacity via knife development, e.g., ball-tipped Flushknife [29], also may help. Fundamentally, if operators had endoscopes that would allow surgical type triangulation and tissue retraction, the procedure might become much faster and technically simpler. Such devices are being developed for NOTES procedures and may soon be available to support ESD.

## Conclusions

In this large series performed by a single, experienced operator, ESD was associated with high curative resection rates and low complication rates in the short term across both upper and lower gastrointestinal tracts. Disease-specific and overall long-term prognoses were excellent after curative resection of criteria lesions. These data could help clinicians to choose ESD as curative but less radical treatment for the gastrointestinal tract, not only in guideline but also in expanded criteria.

## Acronyms

ESD: Endoscopic submucosal dissection
EMR: Endoscopic mucosal resection
UMIN-CTR: University Hospital Medical Information Network-Clinical Trials Registry
ade: Adenoma
m: Mucosal
sm: Submucosal
ca: Cancer
diff.: Differentiated
ly: Lymphatic invasion
v: Vascular invasion
UL: Ulceration
LGIN: Low grade intraepithelial dysplasia
HGIN: High grade intraepithelial dysplasia
EP: Epithelium
LPM: Lamina propria mucosae
MM: Muscularis mucosa
inf α: Infiltrative growth pattern, expansive type
CI: Confidence interval
